# Incident Cancer Risk in Patients with Incident Type 2 Diabetes Mellitus in Hungary (Part 1)

**DOI:** 10.3390/cancers16091745

**Published:** 2024-04-29

**Authors:** Zsolt Abonyi-Tóth, György Rokszin, Ibolya Fábián, Zoltán Kiss, György Jermendy, Péter Kempler, Csaba Lengyel, István Wittmann, Gergő A. Molnár, Gábor Sütő

**Affiliations:** 1 RxTarget Ltd., 5000 Szolnok, Hungary; abonyi-toth.zsolt@rxtarget.hu (Z.A.-T.); rokszin.gyorgy@rxtarget.hu (G.R.); fabian.ibolya@rxtarget.hu (I.F.); 2 Department of Biostatistics, University of Veterinary Medicine, 1078 Budapest, Hungary; 3 Second Department of Medicine and Nephrology-Diabetes Centre, University of Pécs Medical School, 7624 Pécs, Hungary; zoltan_kiss2@merck.com (Z.K.); molnar.gergo@pte.hu (G.A.M.); suto.gabor@pte.hu (G.S.); 4 Department of Internal Medicine, Bajcsy-Zsilinszky Hospital, 1106 Budapest, Hungary; gyjermendy@bajcsy.hu; 5 Department of Medicine and Oncology, Faculty of Medicine, Semmelweis University, 1089 Budapest, Hungary; kempler.peter@med.semmelweis-univ.hu; 6 Department of Internal Medicine, University of Szeged, 6720 Szeged, Hungary; lengyel.csaba@med.u-szeged.hu

**Keywords:** type 2 diabetes mellitus, cancer, epidemiology, new-onset diabetes

## Abstract

**Simple Summary:**

Newly diagnosed type 2 diabetes mellitus is associated with a higher risk of cancer, possibly in young individuals with diabetes. While the incidence of cancer is mildly decreasing in the background population, it does not decrease in patients with type 2 diabetes. In non-diabetic persons, certain types of cancer also occur in young age, while the 60–69-year-old group dominates for cancer cases in patients with type 2 diabetes. The majority of the new cancer cases is recognized within 6 months after the diagnosis of diabetes. This may call for increased cancer surveillance in patients with newly diagnosed type 2 diabetes mellitus.

**Abstract:**

(1) Background: Patients with type 2 diabetes mellitus (T2DM) are at higher risk of cancer but how these two diseases associate is still debated. The goal of this study was the assessment of the overall incidence of cancer among patients with newly diagnosed T2DM in Hungary. (2) Methods: A nationwide, retrospective, longitudinal study was performed using a Hungarian database. After exclusion of cases of age < 18 years, with gestational diabetes, with polycystic ovary syndrome, and with type 1 and prevalent type 2 diabetes mellitus, the incident T2DM (approx. 50,000 cases yearly) and for comparison, the diabetes-free Hungarian adult population (approx. 7,000,000 cases yearly) was included in the study. The primary endpoints were the overall and site-specific incidence and annual percentage change of the incidence of cancer in both populations. (3) Results: The overall incidence of cancer in patients amounted to 29.4/1000 and 6.6/1000 with or without T2DM, respectively, and the OR (95%CI) of cancer of the T2DM group was 4.32 (4.14–4.53), *p* < 0.0001. The risk of having cancer was age dependent. The incidence of cancer was declining in the non-diabetic but was unchanged in the T2DM population. The average lag time of diagnosing cancer after the detection of T2DM was 3.86 months. (4) Conclusions: Incident T2DM is associated with a significantly higher overall risk of incident cancer, with a reverse correlation of age. Newly registered T2DM patients were suggested to be screened for cancer within 6 months.

## 1. Introduction

Type 2 diabetes mellitus (T2DM) is associated with vascular complications [1], however, an increased incidence of cancer can also be observed. A review reported an association between diabetes mellitus (DM) and cancer with overall relative risk of 1.10 (1.01–1.17) in patients with DM (including Type 1 DM) versus non-DM subjects [2]. Patients with T2DM have an underlying enhanced risk of liver, pancreas, endometrium, colon, rectum, breast, and bladder cancer, and are at a decreased risk for prostate cancer [2,3].

The burden of cancer seems to show an increased rate worldwide; however, the different geographical regions are not equally influenced [4]. An annual decrease in the incidence of cancer was estimated in Hungary within the range of 0 to −0.9% [4], but there are no measured data for patients with T2DM patients or for the non-diabetic population.

Cancer and T2DM share a lot of risk factors [3]. Glucotoxicity, hyperinsulinemia, inflammation, and oxidative stress [5] are major contributors of the development of long-term complications in T2DM. Moreover, obesity further contributes to the development of both T2DM and cancer. Alterations of leptin [6,7] and adiponectin [8,9] in T2DM may also play a role in the development of cancer.

Most of the data regarding T2DM and cancer are available for prevalent cases of T2DM when there is a longer duration of T2DM [10]. Data related to the risk of cancer in patients with newly diagnosed T2DM are scarcer [11,12]. The available data suggest that immediately after diagnosis, the incidence of cancer is high; it sharply decreases during the first year, reaches a nadir, then reaches a plateau, or it increases again for several types of cancer [13,14].

Temporal analysis of the incidence of cancer in recently discovered T2DM may provide insight into many inherent properties of these diseases. Early onset of cancer after diagnosis of T2DM may be the consequence of the unknown onset of T2DM, since the onset of the disease may precede the diagnosis by months or years [15]. Moreover, the abovementioned processes promoting cancer may be initiated already during prediabetes, as indicated by data showing an increased incidence of cancer in prediabetes [16].

In the present study, we focused on the development of cancer in incident, i.e., relative, newly diagnosed cases of T2DM, as data are still somewhat lacking for these patients. The short duration of T2DM also has the advantage of ruling out the effect of antidiabetic medications on cancer development. The odds of developing cancer could be compared to a large control population. Our paper was intended to simultaneously provide data on the role of diabetes, gender, and age, as well as to analyze temporal trends and provide site-specific data for all the major cancer types with a nationwide coverage. This would be a unique approach, according to our knowledge.

## 2. Materials and Methods

**Figure 1:**
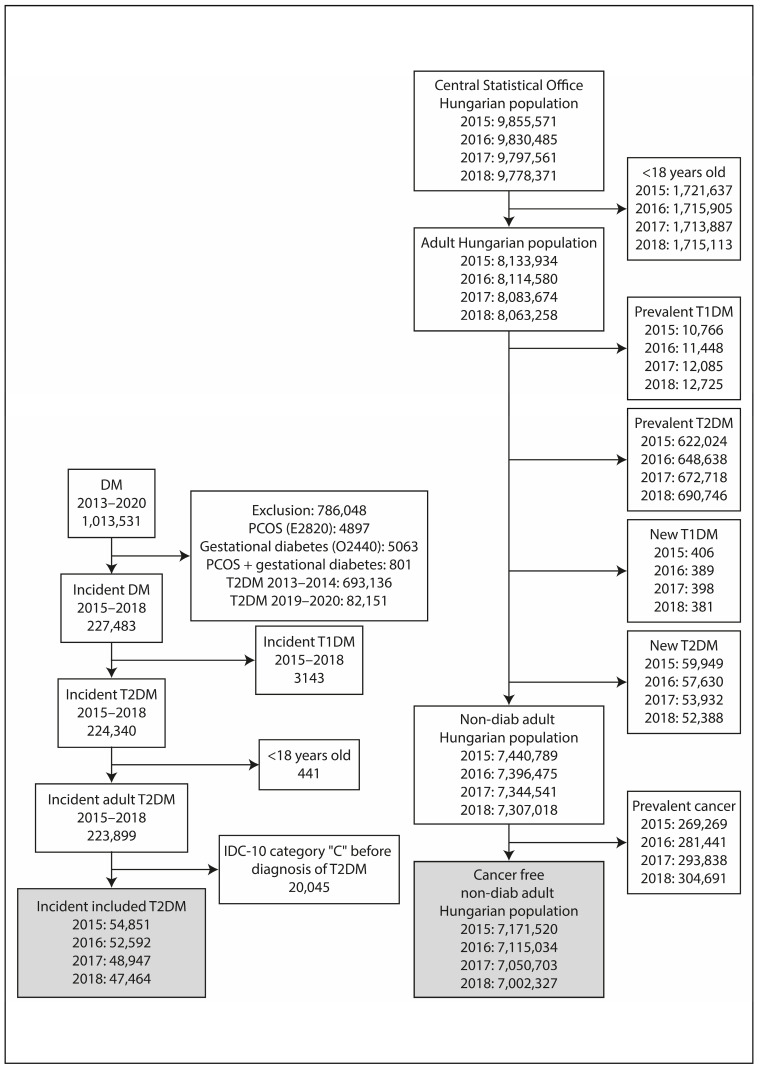
Flowchart of the study. Patients diagnosed with or without T2DM between 2015–2018 were screened for incident cancer up to 1 year after the onset of DM. Patients with ICD-10 code “C” before the diagnosis of T2DM were excluded.

A nationwide, retrospective, longitudinal study was performed using data from two different sources. The epidemiological data of adult T2DM patients were obtained from the National Institute of Health Insurance Fund database (NHIF). Similar data of the adult non-diabetic population were gathered from the database of the Hungarian Central Statistical Office (HCSO). Aiming to identify new cancer patients among incident T2DM patients and non-diabetic adult Hungarian population, a screening algorithm was developed (Figure 1). Inclusion criteria were age > 18 years and T2DM for the cases and age > 18 years and the absence of T2DM for the controls. Patients with any prior cancer between 2009 and 2014 were excluded from the analysis.

### 2.1. Incident T2DM in the Adult Population

The nationwide insurance system covers the health care data of nearly 100% of the Hungarian population and collects information of ICD-10 code from the in- and out-patient visits. The data were anonymized. The index period covered all patients older than 18 years who were diagnosed with T2DM between 2015 and 2018. The presence of ICD-10 code C was searched during the index period, from 1st of January 2009 and one year after the date of diagnosis of T2DM. Only incident T2DM patients were included in the study (Figure 1). The NHIF has only provided aggregate data for subgroups with a number of cases more than 10 and the results of analysis models, in line with data protection rules.

### 2.2. Non-Diabetic Adult Hungarian Population

The HCSO collects all health-related data from the entire Hungarian population. Patients younger than 18 years who were diagnosed with prevalent or recent-onset T2DM or T1DM were excluded from the database in each year between 2015 and 2018 (Figure 1). The remaining part of the population represented the non-diabetic adult population of Hungary. The presence of ICD-10 code C was searched to identify incident cancers. In the present analysis, the International Classification of Diseases, 10th Revision (ICD-10) codes between C00 and C99 have been used. The details are given in the supplement (Supplementary Methods).

### 2.3. Statistical Analysis

R version 4.0.4 was used for a binomial logistic regression involving the population without cancer and T2DM on 1st of January each year. The index date was either the diagnosis of T2DM (incident cases) or the 1st of January for the others. The dependent variable was the incidence of cancer, while the independent variables were the presence of T2DM, age group, gender, and all their interactions. The follow-up period was 365 days for everyone.

The odds ratio (OR) and average annual percent change (AAPC) were calculated by bootstrap with one billion repetitions and are shown together with their 95% confidence intervals. An adjustment for age and gender was made in statistical analyses, using a mean number of the underlying population from each subgroup throughout the study period.

The study protocol was approved by the Ethical Board of the University of Pécs (9326-PTE 2022), by the National Institute of Pharmacy and Nutrition (KRID: 641936355) and by Medical Research Council (BMEÜ/95-3/2022/EKU).

## 3. Results

### 3.1. Selection of Patients

A total of 1,013,531 patients with incident DM were found between 2013 and 2020 (Figure 1). A population of 786,048 patients was excluded due to the presence of polycystic ovary syndrome or gestational diabetes or both, or diagnosis of DM between 2013–2014 or 2019–2020. In addition, 441 were excluded because of an age below 18 years, while 3.143 had T1DM. Patients with a diagnosis of cancer before the diagnosis of T2DM (*n* = 20,045) were also excluded. Finally, 54,851, 52,592, 48,947, and 47,464 patients with incident T2DM remained to fulfill inclusion criteria in the years 2015, 2016, 2017, and 2018, respectively.

The population of Hungary was 9,855,571, 9,830,485, 9,797,561, 9,778,371 in the years of 2015, 2016, 2017, and 2018, respectively. After the exclusion of persons with an age below 18 years, finally, 7,440,789, 7,396,475, 7,344,541, and 7,307,018 patients free of diabetes were identified for those years.

### 3.2. The Incidence of Cancer

**Figure 2:**
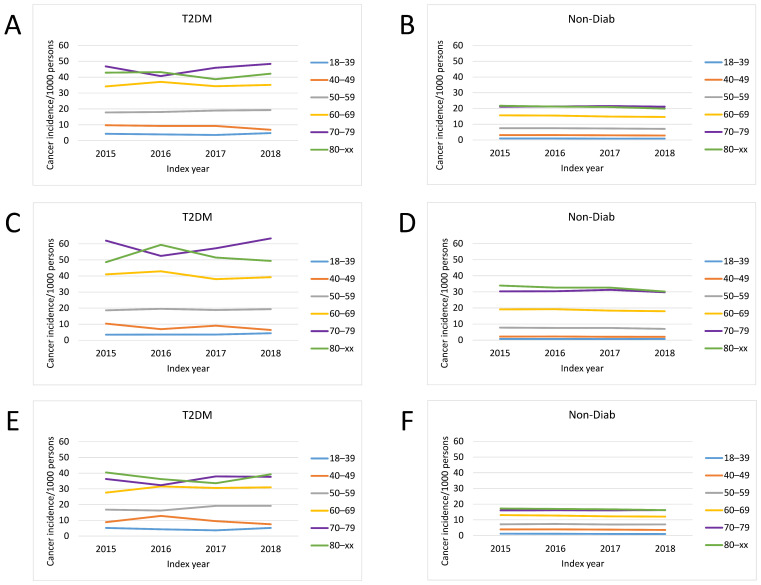
Incidence of developing cancer in different age groups: (**A**,**B**) in the total population, (**C**,**D**) in male persons, (**E**,**F**) in females, (**A**,**C**,**E**) in patients with T2DM. (**B**,**D**,**F**) show data related to the Non-Diab group.

The overall incidence of a new cancer was markedly higher in patients with T2DM than it was in non-diabetic persons (Figure 2). The incidence of cancer was age dependent, being the lowest in the 18–39 age group and the highest in the 70–79 and 80+ age groups in both genders (Figure 2).

**Figure 3:**
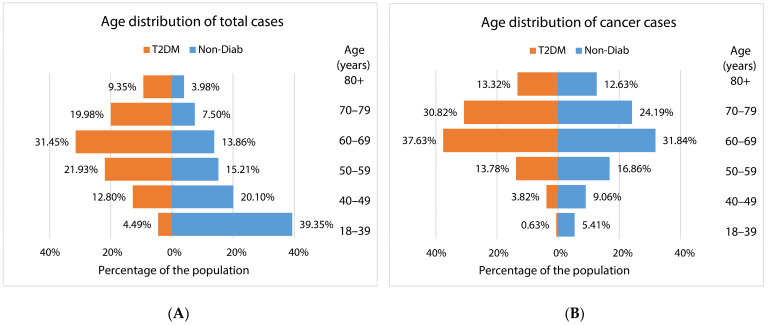
Age distribution of (**A**) the populations at risk and (**B**) incident cancer cases in patients with type 2 diabetes mellitus and in nondiabetic controls.

The age distribution of patients at risk and those with incident cancer showed a different pattern in patients with T2DM compared to the Non-Diab group (Figure 3A). The proportion of 18–39-year-old patients was highest in the Non-Diab group among total cases. However, among patients with T2DM but without cancer, the 60–69-year-old group dominated (Figure 3A). By contrast, among patients with cancer, the 60–69-year-old group dominated both in patients with and without T2DM (Figure 3B).

### 3.3. Risk of Cancer

**Figure 4:**
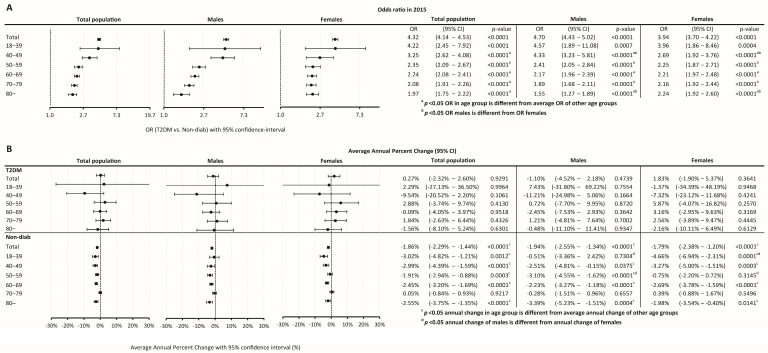
(**A**) The odds ratio of developing cancer in different age groups and (**B**) the annual change of incidence between 2015 and 2018.

The odds ratio (OR) of having cancer in the T2DM group as compared to the Non-Diab group was 4.32 (4.14–4.53), *p* < 0.0001) in the total population, while it was 4.70 (4.43–5.02), *p* < 0.0001) and 3.94 (3.70–4.22), *p* < 0.0001) in males and females, respectively (Figure 4A).

When investigating different age groups, we found that the odds were highest in the 18–39-year-old subgroup in the T2DM population, both in males and females. On the other hand, the lowest OR was present in the 80+ age group in the overall population, both in males and females. Male compared to female persons had higher odds of having cancer between 40–49 years. Females had higher odds of having an incident cancer in the 80+ age group as compared to males (Figure 4A).

Temporary trends were also analyzed, and it was found that in the Non-Diab group, between 2015–2018, overall AAPC decreased by −1.86% (−2.29–−1.44%, *p* < 0.0001) annually, as opposed to cancer incidence in the T2DM group, which was practically unchanged (AAPC, 0.27 [−2.32–+2.60, *p* = 0.9291]) (Figure 4B). A similar AAPC difference was found between the T2DM and Non-Diab groups in males and females (Figure 4B).

### 3.4. Risk of the Incidence of Cancer According to Specific Sites

**Figure 5:**
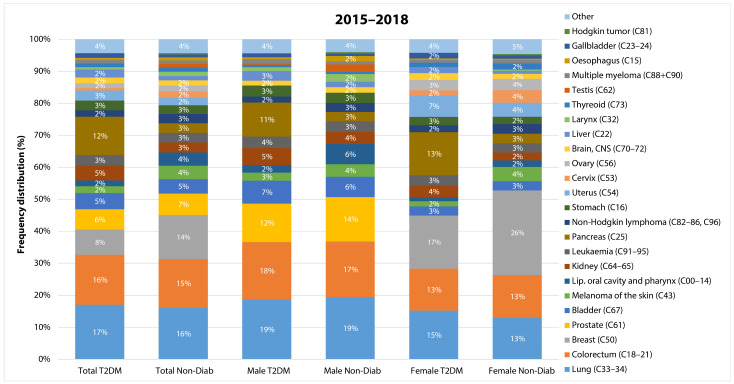
The most common cancer sites in non-diabetic controls and in patients with type 2 diabetes mellitus as a percentage of total cancer cases in the total population, in male patients and in female patients.

We also analyzed the risk of individual cancer sites. The most common manifestations of cancer were lung, colorectal, breast, prostate, and bladder cancers in both groups. Cancers of the kidney, pancreas, and uterine corpus seemed to account for a larger proportion of patients in the T2DM as opposed to the Non-Diab group (Figure 5).

**Figure 6:**
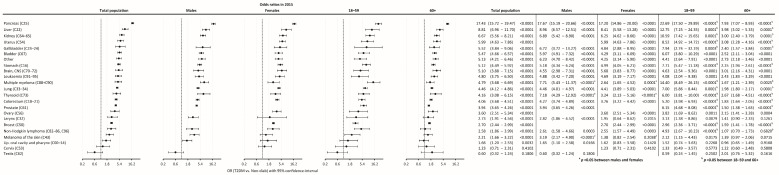
The odds ratio of developing cancer at different sites in the total population in 2015, in males, in females, in the age groups of 18–59 years, and in the age group of 60+ years.

The highest odds of cancer were found for pancreas, liver, kidney, uterus, gallbladder, bladder, stomach, brain/CNS, leukemia, multiple myeloma, lung, thyroid, colorectum, prostate, ovary, larynx, breast, non-Hodgkin lymphoma, and melanoma of the skin in T2DM patients compared to the non-diabetic population (Figure 6). Pancreatic cancer had the highest odds (OR: 17.43). Cancer odds, in general, showed similar trends in male and female patients, whereas multiple myeloma, thyroid cancer, and melanoma had a stronger association with T2DM in males than in females. Several types of cancer showed a higher risk in the 18–59 vs. the 60+ age groups, including pancreas, liver, kidney, uterus, lung, colorectal, prostate, and ovarian cancers.

### 3.5. Annual Average Percentage Changes (AAPC)

**Figure 7:**
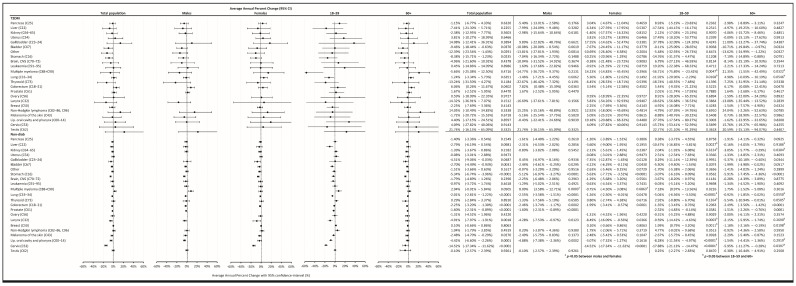
AAPC of developing cancer at different sites in the total population, in males, in females, in the age group 18–59 years, and in the age group of 60+ years.

In the Non-Diab population, the overall incidence of gallbladder, bladder, stomach, colorectal, prostate, larynx, oropharyngeal, and cervix cancer declined between 2015–2018, whereas in the T2DM group, the incidence decreased only in cases of bladder and stomach. Interestingly, in the T2DM group, the incidence of colorectal cancer significantly increased. Female and male patients showed similar trends in both groups, except for multiple myeloma, which showed an increased rate in males but not in females. We could find a significant difference between the 18–59 and 60+ age groups for several cancers. In some cases, the direction of change was the opposite; for instance, for breast cancer in the Non-Diab group, the frequency increased in younger but decreased in older subjects (Figure 7).

### 3.6. Age Distribution

**Figure 8:**
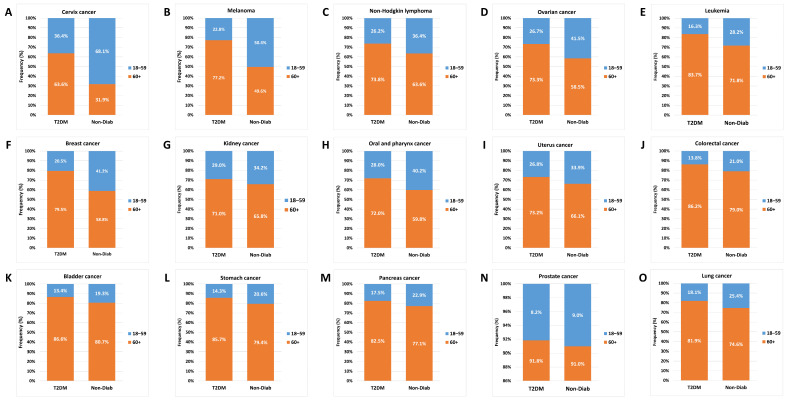
Age distribution of site-specific incident cancers in non-diabetic controls and in patients with type 2 diabetes mellitus. Graphs (**A**–**O**) denote different cancer sites, as seen in individual graph headings.

Comparing the age distribution for individual cancer types, we found that the contribution of the 60+ age group was similar for lung, pancreas, stomach, bladder, colorectal, uterine corpus, and kidney cancer, but with a dominance of the T2DM group. By contrast, in the case of cervix, melanoma, non-Hodgkin lymphoma, ovarian, and breast cancers, the contribution of the 18–59-year-old group was higher in the Non-Diab group than in the T2DM group (Figure 8).

### 3.7. Time of Detection

**Figure 9:**
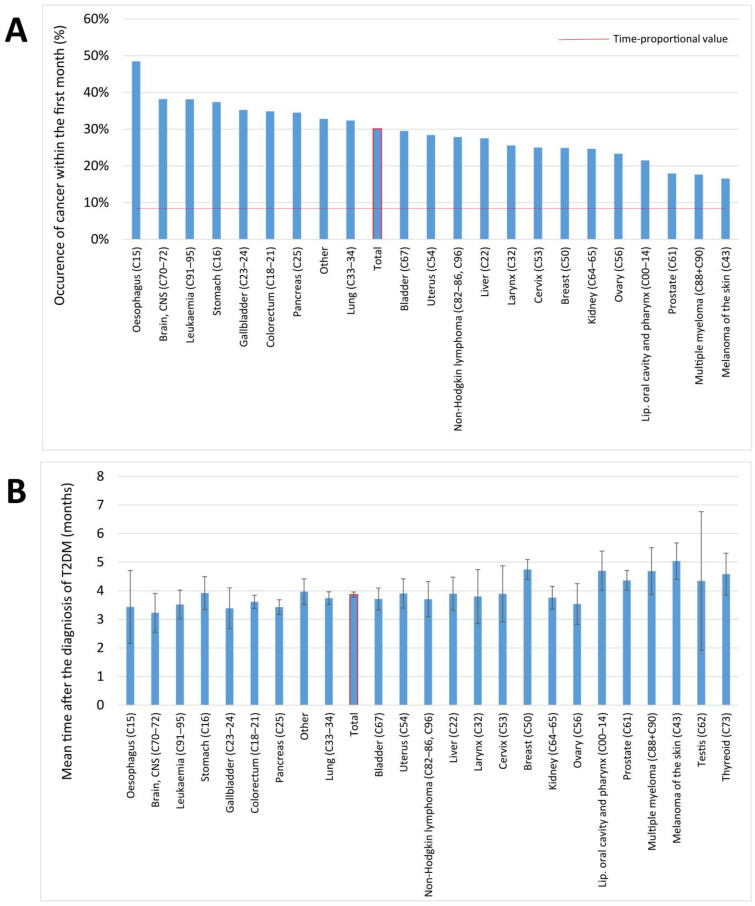
(**A**) Occurrence of cancer in the first month and (**B**) the average lag time of the detection of a cancer after the diagnosis of T2DM. The red line in panel (**A**) denotes 8.33%, that is the chance of getting cancer in the first month within the observation year, if it was by chance only (1/12).

We further analyzed cancer onset within the first year. Overall, 30% of all cancers occurred in the first month after the diagnosis of T2DM. For individual cancer types, the percentage varied between 16.5–48.5%, and the highest proportion was observed in the case of esophagus, brain, leukemia, stomach, gallbladder, colorectal, and pancreas cancers. The average lag time between diagnosis of T2DM and diagnosis of overall cancer was 3.86 months, and the average lag time was below the expected 6 months for all of the cancer types investigated (Figure 9).

## 4. Discussion

The overall and site-specific incidence and AAPC of incident cancer within a year after the diagnosis of T2DM was analyzed in our study between 2015 and 2018. The analysis was stratified by gender and age, while the whole non-diabetic Hungarian population served as control. To our knowledge, this is the first analysis performed on the change of cancer incidence in recently diagnosed T2DM over four consecutive years.

In our study, the overall incidence of any cancer was much higher among patients with incident T2DM vs. Non-Diab persons (32.11 vs. 6.75/1000 persons). The overall odds of developing a cancer were 4.32-times higher in T2DM patients compared to the Non-Diab controls.

The association between the development of cancer and T2DM has already been confirmed in numerous epidemiological observations (reviewed in [17]); however, this is mainly so for prevalent DM. Several links [18] have been described between T2DM and cancer, such as obesity, hyperglycemia, dyslipidemia, oxidative stress, inflammation, and hyperinsulinemia [17]. Much less understood is the association between the incidence of cancer and recent-onset T2DM; however, some evidence also exists concerning this association [12].

With regard to the temporal trends of incident cancers, the highest incidence of cancer was found at the time of diagnosis of T2DM, and a decrease was detected within the first year after diagnosis of DM in two studies [13,14]. In both papers, the incidence of cancer was the lowest between 2 and 3 years after the diagnosis of DM; it increased again thereafter, and showed a second peak around 8 years [14], or 5–8 years in another paper [19].

According to another observation, the risk of cancer was highest within the first six months after diagnosis of DM (HR: 2.03 [1.99–2.03]), and decreased in the time interval of 6 months–3 years (HR: 1.19 [1.18–1.21]. The risk was significantly elevated for all 17 sites of cancer, but it became non-significant after 6 months for esophagus, larynx, breast, cervical, and ovarian cancer [11].

A further study compared the periods of 0–3 months and 3 months–10 years after diagnosis of DM, and a significant, approximately 2–3 times higher, risk was found for colorectal, lung, cervical, endometrial, ovarian, and prostate cancer, but not for breast or thyroid cancers within the first 3 months. In the period after 3 months, the significance diminished for lung, cervical, and ovarian cancers [12].

Another paper presented similar results, but found an increased risk of cancers of the pancreas, colorectum, lung, endometrium, prostate, thyroid, and bladder, but not for the cervix or ovaries in the first 3 months. The highest HR was present for cancer of the pancreas (8.13 [5.42–12.20]) [20]. We found an increased risk for nearly all types of cancer, except for cervical and testicular cancer. We have also found that the risk was highest for cancer of the pancreas.

One striking finding of our study was the high occurrence of cancer immediately after the diagnosis of T2DM. Some data indicate that the development of cancer may in fact even precede the diagnosis of DM [20]. Therefore, in our study, cases with cancer diagnosed before the index date were excluded. However, hyperglycemia-related metabolic changes that promote carcinogenesis may be initiated already in prediabetic conditions and this notion is supported by ample evidence in the literature [15,16,21,22,23,24,25,26,27,28].

However, reverse causality in the background cannot be excluded, mainly in the case of cancer of the pancreas, which may directly damage insulin-secreting beta cells and impair their function or induce peripheral insulin resistance, thereby causing DM [13]. The latter mechanism cannot be excluded even in the case of extrapancreatic cancers [29].

On the other hand, the high incidence of cancer in the months just following the diagnosis of T2DM might also be in part the consequence of a detection bias or ascertainment bias, according to the literature [30]. This term means that at the time of diagnosing T2DM, more diagnostic tests are being carried out as a part of routine diagnostic schemes, and cancer is diagnosed by chance during such routine tests. While this approach may appear problematic in epidemiological studies, we believe that this might be an important practical consideration. Namely, at the diagnosis of T2DM, or within a 6 months-period after, it might be worth carrying out at least basic diagnostic procedures in terms of cancer screening (such as abdominal ultrasound, chest X-ray or low-dose CT, fecal occult blood tests, mammography, and gynecological testing or PSA screening).

A 2010 consensus statement suggests that “patients with diabetes should … undergo appropriate cancer screenings as recommended for all people in their age and sex” [3]. However, our data might also support the opinion of Suh et al., who suggest that in order to enable early diagnosis and primary prevention, “cancer should be screened in routine diabetes assessment” [31].

Concerning the relation of colorectal cancer and new-onset T2DM, a study found that the HR of cancer was numerically higher in patients aged < 55 years as compared to the total population (1.8 [1.1–2.9] vs. 1.4 [1.3–1.6], respectively) [32]. In our study, we observed a high OR of cancer of the 18–39-year-old group with incident T2DM, although the lower number of cases and wide confidence interval somewhat limits generalizability of this finding. Nevertheless, the potential excess risk in young individuals with T2DM may also be important because of rising proportion of young individuals among patients with T2DM [33,34,35,36]. This pattern of risk in young individuals is very similar to the incidence of cardiovascular morbidity and mortality in T2DM patients, being higher in young-onset diabetes [37,38].

We found that the incidence of cancer did not change among diabetic patients between 2015–2018, but slightly declined in the non-diabetic population. The latter is in line with the estimated annualized rate change of age-standardized incidence rate of cancers excluding non-melanoma skin cancer, which was globally between 0 and −0.2% (95% UI,−0.9% to 0.5%) and in Hungary between 0 and −0.9% in 2010–2019 [4]. In contrast, the unchanged rate of incident cancer among recently diagnosed T2DM patients suggests that diabetes itself may contribute to the sustained cancer incidence.

The major strength of our study is the very high coverage of the Hungarian population by the database of NHIF and HCSO. The report of the date and histological diagnosis of a cancer is mandatory in this collection of data. The first report of T2DM may also be recovered. This fact makes the database of our analysis valid and accurate.

The weakness of data collection is that, due to data protection regulations of NHIF, it is only able to provide aggregated data, i.e., there are no available data for subgroups including less than 10 patients. A further weakness is that laboratory data regarding T2DM follow-up and medical treatment were not available for analysis. A low number of cases and events in distinct age groups may also be a limitation.

## 5. Conclusions

In summary, incident T2DM associates with an enhanced risk for incident cancer, the overall risk being as high as 4.45. There are 18 cancer sites where the risk of cancer is higher in diabetic patients, with the pancreas, liver, and kidney bearing the highest risk of developing cancer. The risk is substantially higher in patients of younger ages, where the cardiovascular risk is also enhanced. The incidence of cancer is slowly declining in the non-diabetic population but not among diabetic patients. The age, gender, and site-specific differences among different cancer sites need further exploration. We might need to re-evaluate the current screening strategies in terms of cancer in (young) individuals with newly registered T2DM.

## Data Availability

Data generated during the study are available upon reasonable request from the corresponding author. Individual, patient-level data are not available due to data protection rules of the NHIF.

## Supplementary Materials

The following supporting information can be downloaded at: https://www.mdpi.com/article/10.3390/cancers16091745/s1, Supplementary methods.
