# Supplementary material for: Incident Cancer Risk in Patients with Incident Type 2 Diabetes Mellitus in Hungary (Part 1)
Source: Cancers (Basel). 2024 Apr 29;16(9):1745. doi: 10.3390/cancers16091745 (PMC11083545; doi:10.3390/cancers16091745)
Supplement: Supplementary file 1 [file cancers-16-01745-s001.zip › cancers-2897677-supplementary/Supplements.pdf]

SUPPLEMENTARY METHODS

Methods of identification of cancer and T2DM cases

For the cancer cases, we considered ICD Codes starting with C, except for C44 and C77-80. We used Ferlay's definition to group the ICD codes to determine the cancer sites17. In the case of multiple cancers, we considered only the sites having the majority of ICD codes and ignored all the others. The supposed date of diagnosis was the occurrence of the first ICD code in in- or outpatient records for patients who had at least two records with different date of the same cancer. Data was available between 1st January 2009 and 31st December 2022.

For T2DM cases, we considered ICD-10 E10-14 codes in in- and outpatient and drug refill records. We included those patients who had ICD codes followed by at least 30 and at most 365 days by another ICD code in the records to avoid supposed but not proved cases. The date of diagnosis was the date of the first ICD code meeting these criteria. We also included patients who died in 60 days after the occurrence of the diabetes ICD code. Finally, we also included patients having at least two refills with ATC A10 independently of ICD codes. We did not consider diabetic patients who had any polycystic ovary syndrome (ICD E28.2) or gestational DM (O24.4) code. Data was available between 1st January 2013 and 31st December 2020.

1. Because of possible coding errors we used a hierarchical definition to separate T1DM and T2DM cases. We considered patients having T1DM if (main rule): they had E10 code, the number of E10 codes were equal to or more than the number of E11 codes, they had insulin refills and they had no OAD (oral antidiabetics) refill over 180 days of the first insulin refill.

2. (some irregular OAD): they had E10 code, the number of E10 codes were equal to or more than the number of E11 codes, they had insulin refills and they had at most three of OAD refill over 180 days of the first insulin refill, but at least 6 of insulin refills

3. (without ICD): they had no E10 nor E11 code, they had insulin refills and had at most three of OAD refills over 180 days of the first insulin refill, but at least 6 of insulin refills, age was <=35 at first refill

4. (some OAD combination): they had E10 code, the number of E10 codes were more or equal to the number of E11 codes, their first refill was insulin and there were only insulin refills during the first 180 days; then there were OAD refills with an insulin refill in the 7 days neighborhood of each

5. (some OAD): they had E10 code, the number of E10 codes were more or equal to the number of E11 codes, their first refill was insulin and there were only insulin refills during the first 180 days, and further OAD refills without an insulin refill in the 7 days neighborhood of each, but the last refill was insulin again

6. (some OAD and combination): they had E10 code; the number of E10 codes were more or equal to the number of E11 codes, their first refill was insulin and there were only insulin refills during the first 180 days, further, there were OAD refills with or without

an insulin refill in the 7 days neighborhood of each, but the last refill was insulin or OAD+insulin in 7 days again

We considered patients having T2DM in case they did not meet the above criteria or were at least 40 years old at diagnosis.

r code

```
library(RODBC)

channel=odbcDriverConnect("DSN=xxx;uid=xxx;pwd=xxx")

setwd("d:/result")


write.table(NULL, "groups.csv")

write.table(NULL, "years.csv")


library(dplyr)


# People at risk


atRisk <- data.frame(

  gender =
c("male","male","male","male","male","male","male","male","male","male","male","male","male",
"male","male","male","female","female","female","female","female","female","female","female","f
emale","female","female","female","female","female","female","female"),

  group = c("T2DM","T2DM","T2DM","T2DM","T2DM","T2DM","T2DM","T2DM","Non-
diab","Non-diab","Non-diab","Non-diab","Non-diab","Non-diab","Non-diab","Non-
diab","T2DM","T2DM","T2DM","T2DM","T2DM","T2DM","T2DM","T2DM","Non-diab","Non-
diab","Non-diab","Non-diab","Non-diab","Non-diab","Non-diab","Non-diab"),

  age = c("0-59","0-59","0-59","0-59","60-xx","60-xx","60-xx","60-xx","0-59","0-59","0-59","0-
59","60-xx","60-xx","60-xx","60-xx","0-59","0-59","0-59","0-59","60-xx","60-xx","60-xx","60-
xx","0-59","0-59","0-59","0-59","60-xx","60-xx","60-xx","60-xx"),

  year =
c(2015,2016,2017,2018,2015,2016,2017,2018,2015,2016,2017,2018,2015,2016,2017,2018,2015,20
16,2017,2018,2015,2016,2017,2018,2015,2016,2017,2018,2015,2016,2017,2018),

  nrisk =
c(11673,11366,10791,10648,14528,13984,13011,12467,2696402,2668260,2637402,2624293,6841
63,691764,695900,692153,9384,8875,8584,8620,19266,18367,16561,15729,2690854,2649552,260
7865,2582551,1100101,1105458,1109536,1103330)

)

atRisk$gr <- paste(atRisk$gender, atRisk$age, atRisk$group, sep='')


# Number at risk in the cohorts
```

```
nf1 <- sum(atRisk[atRisk$group == "Non-diab" & atRisk$age == "0-59" & atRisk$gender ==
"female", ]$nrisk)

nf2 <- sum(atRisk[atRisk$group == "T2DM" & atRisk$age == "0-59" & atRisk$gender ==
"female", ]$nrisk)

nf3 <- sum(atRisk[atRisk$group == "Non-diab" & atRisk$age == "60-xx" & atRisk$gender ==
"female", ]$nrisk)

nf4 <- sum(atRisk[atRisk$group == "T2DM" & atRisk$age == "60-xx" & atRisk$gender ==
"female", ]$nrisk)


nm1 <- sum(atRisk[atRisk$group == "Non-diab" & atRisk$age == "0-59" & atRisk$gender ==
"male", ]$nrisk)

nm2 <- sum(atRisk[atRisk$group == "T2DM" & atRisk$age == "0-59" & atRisk$gender ==
"male", ]$nrisk)

nm3 <- sum(atRisk[atRisk$group == "Non-diab" & atRisk$age == "60-xx" & atRisk$gender ==
"male", ]$nrisk)

nm4 <- sum(atRisk[atRisk$group == "T2DM" & atRisk$age == "60-xx" & atRisk$gender ==
"male", ]$nrisk)


# List of cancers
cancers  <- c(
  "Bladder (C67)",
  "Brain, CNS (C70-72)",
  "Colorectum (C18-21)",
  "Gallbladder (C23-24)",
  "Kidney (C64-65)",
  "Larynx (C32)",
  "Leukaemia (C91-95)",
  "Lip. oral cavity and pharynx (C00-14)",
  "Liver (C22)",
  "Lung (C33-34)",
  "Melanoma of the skin (C43)",
  "Multiple myeloma (C88+C90)",
  "Non-Hodgkin lymphoma (C82-86, C96)",
```

```
  "Other",
  "Pancreas (C25)",
  "Stomach (C16)",
  "Thyreoid (C73)",
  "Uterus (C54)",
  "Ovary (C56)",
  "Breast (C50)",
  "Cervix (C53)",
  "Prostate (C61)",
  "Testis (C62)",
  "Hodgkin tumor (C81)",
  "Oesophagus (C15)"
)


# Main function for the computations
# The model is like this: glm(cbind(event, nrisk) ~ gr + gr:year2 - 1, data = ds, family =
"quasibinomial")


# Problem: The model works on logit scale. If we calculate marginal (e.g. males all ages) estimates
# and confidence intervals, they are not valid on probability scale.
# We need to calculate them directly on the probability scale.
process_model <- function(model) {try({
  set.seed(314)
  repetitions <- 1000000
  # The model coefficients are random variables. We generate different estimations
  # using the coefficients' estimation and covariance.
  betas <- MASS::mvrnorm(repetitions, summary(model)$coef[, 1], vcov(model) )
  # The first row contains the model's point estimations
  betas[1, ] <- t(summary(model)$coef[, 1])
  # Column 9-16 contained the annual change estimate. Now they contain the estimate in the second
year.
```

```
for (i in 1:8) {
  betas[, 8 + i] <- betas[, 8 + i] + betas[, i]
}
# We go to probability scale. The colums contain the probability estimate for distinct subgoups.
responses <- na.omit(exp(betas) / (1 + exp(betas)))
repetitions <- nrow(responses)

resp <- responses[, 1:8]

# Vectors to store the results
contrast <- character(15)
or <- numeric(15)
cilwr <- numeric(15)
ciupr <- numeric(15)
pvalue <- numeric(15)
reps <- numeric(15)
ormatrix <- matrix(NA, nrow = repetitions, ncol = 9)

# Calculates OR estimates
# i: row number in the vectors to store the results
# resp: probabilities in subgroups
# repetitions: number of repetitions
# name: contrast name in the result file
# c1: weights for marginal1
# c2: weights for margonal2
calculateOR <- function(i, resp, repetitions, name, c1, c2) {
  # Estimated probability for marginal1
      matrix <- matrix(rep(c1, repetitions), nrow = repetitions, byrow = T)
  x1 <- rowSums(resp * matrix)
  # Estimated probability for marginal2
```

```
  matrix <- matrix(rep(c2, repetitions), nrow = repetitions, byrow = T)
  x2 <- rowSums(resp * matrix)
      # OR
  oddsratios <- (x1 / (1 - x1)) / (x2 / (1 - x2))
  ormatrix[, i] <<- oddsratios
  contrast[i] <<- name
  or[i] <<- oddsratios[1]
      # Confidence interval and p-value
  cilwr[i] <<- quantile(oddsratios, probs = 0.025, na.rm = T)
  ciupr[i] <<- quantile(oddsratios, probs = 0.975, na.rm = T)
  reps[i] <<- sum(!is.na(oddsratios))
  pvalue[i] <<- min(sum(oddsratios < 1), sum(oddsratios > 1)) * 2 / reps[i]
}

# Calculate ORRs between genders for different age grouops
# i: row number in the vectors to store the results
# index: number of age group
# ormatrix: already calculated ORs T2DM vs. non-diab
# name: contrast name in the result file
compareORgenders <- function(i, index, ormatrix, name) {
  oddsratios <- ormatrix[, index + 3] / ormatrix[, index + 6]
  contrast[i] <<- name
  or[i] <<- oddsratios[1]
  cilwr[i] <<- quantile(oddsratios, probs = 0.025, na.rm = T)
  ciupr[i] <<- quantile(oddsratios, probs = 0.975, na.rm = T)
  reps[i] <<- sum(!is.na(oddsratios))
  pvalue[i] <<- min(sum(oddsratios < 1), sum(oddsratios > 1)) * 2 / reps[i]
}

# Calculate ORRs between age groups
```

```
# i: row number in the vectors to store the results
# resp: probabilities in subgroups
# repetitions: number of repetitions
# name: contrast name in the result file
# ci: weights for marginals (T2DM marginal1, T2DM marginal2, non-diab marginal1 and non-diab marginal2 respectively)
compareORages <- function(i, resp, repetitions, name, c1, c2, c3, c4) {
  matrix <- matrix(rep(c1, repetitions), nrow = repetitions, byrow = T)
  x1 <- rowSums(resp * matrix)
  matrix <- matrix(rep(c2, repetitions), nrow = repetitions, byrow = T)
  x2 <- rowSums(resp * matrix)
  matrix <- matrix(rep(c3, repetitions), nrow = repetitions, byrow = T)
  x3 <- rowSums(resp * matrix)
  matrix <- matrix(rep(c4, repetitions), nrow = repetitions, byrow = T)
  x4 <- rowSums(resp * matrix)
  oddsratios <- ((x1 / (1 - x1)) / (x2 / (1 - x2))) / ((x3 / (1 - x3)) / (x4 / (1 - x4)))
  contrast[i] <<- name
  or[i] <<- oddsratios[1]
  cilwr[i] <<- quantile(oddsratios, probs = 0.025, na.rm = T)
  ciupr[i] <<- quantile(oddsratios, probs = 0.975, na.rm = T)
  reps[i] <<- sum(!is.na(oddsratios))
  pvalue[i] <<- min(sum(oddsratios < 1), sum(oddsratios > 1)) * 2 / reps[i]
}


calculateOR(1, resp, repetitions, "T2DM-Nondiab total", c(0,nf2,0,nf4, 0,nm2,0,nm4)/(nf2+nf4+nm2+nm4), c(nf1,0,nf3,0, nm1,0,nm3,0)/(nf1+nf3+nm1+nm3))
calculateOR(2, resp, repetitions, "T2DM-Nondiab 18-59", c(0,nf2,0,0, 0,nm2,0,0)/(nf2+nm2), c(nf1,0,0,0, nm1,0,0,0)/(nf1+nm1))
calculateOR(3, resp, repetitions, "T2DM-Nondiab 60-xx", c(0,0,0,nf4, 0,0,0,nm4)/(nf4+nm4), c(0,0,nf3,0, 0,0,nm3,0)/(nf3+nm3))
```

```
  calculateOR(4, resp, repetitions, "T2DM-Nondiab total male", c(0,0,0,0, 0,nm2,0,nm4)/(nm2+nm4), c(0,0,0,0, nm1,0,nm3,0)/(nm1+nm3))

  calculateOR(5, resp, repetitions, "T2DM-Nondiab 18-59 male", c(0,0,0,0, 0,1,0,0), c(0,0,0,0, 1,0,0,0))

  calculateOR(6, resp, repetitions, "T2DM-Nondiab 60-xx male", c(0,0,0,0, 0,0,0,1), c(0,0,0,0, 0,0,1,0))


  calculateOR(7, resp, repetitions, "T2DM-Nondiab total female", c(0,nf2,0,nf4, 0,0,0,0)/(nf2+nf4), c(nf1,0,nf3,0, 0,0,0,0)/(nf1+nf3))

  calculateOR(8, resp, repetitions, "T2DM-Nondiab 18-59 female", c(0,1,0,0, 0,0,0,0), c(1,0,0,0, 0,0,0,0))

  calculateOR(9, resp, repetitions, "T2DM-Nondiab 60-xx female", c(0,0,0,1, 0,0,0,0), c(0,0,1,0, 0,0,0,0))


  compareORgenders(10, 1, ormatrix, "T2DM-Nondiab total male-female")

  compareORgenders(11, 2, ormatrix, "T2DM-Nondiab 18-59 male-female")

  compareORgenders(12, 3, ormatrix, "T2DM-Nondiab 60-xx male-female")


  compareORages(13, resp, repetitions, "T2DM-Nondiab 18-59 - 60-xx", c(0,nf2,0,0, 0,nm2,0,0)/(nf2+nm2), c(0,0,0,nf4,  0,0,0,nm4)/(nf4+nm4), c(nf1,0,0,0,  nm1,0,0,0)/(nf1+nm1), c(0,0,nf3,0,  0,0,nm3,0)/(nf3+nm3))

  compareORages(14, resp, repetitions, "T2DM-Nondiab 18-59 - 60-xx male", c(0,0,0,0,  0,1,0,0), c(0,0,0,0,  0,0,0,1), c(0,0,0,0,  1,0,0,0), c(0,0,0,0,  0,0,1,0))

  compareORages(15, resp, repetitions, "T2DM-Nondiab 18-59 - 60-xx female", c(0,1,0,0,  0,0,0,0), c(0,0,0,1,  0,0,0,0), c(1,0,0,0,  0,0,0,0), c(0,0,1,0,  0,0,0,0))


  response <- data.frame(cancer = cancers[cancerNumber], contrast = contrast, OR = or, 'CI lower' = cilwr, 'CI upper' = ciupr, 'p-value' = ifelse(round(pvalue, 4) < 0.0001, "<0.0001", round(pvalue, 4)), repetitions = reps)

  write.table(response, "groups.csv", append = T, col.names = T, row.names = F, sep = ";", dec = ".")


  # annual changes ----


  contrast <- character(39)

  or <- numeric(39)
```

```
cilwr <- numeric(39)
ciupr <- numeric(39)
pvalue <- numeric(39)
reps <- numeric(39)
ormatrix <- matrix(NA, nrow = repetitions, ncol = 18)

calculateAnnualOR <- function(i, responses, repetitions, name, c1) {
  resp1 <- responses[, 9:16] # 1 year later
  resp2 <- responses[, 1:8]  # at start
  matrix <- matrix(rep(c1, repetitions), nrow = repetitions, byrow = T)
  x1 <- rowSums(resp1 * matrix)
  x2 <- rowSums(resp2 * matrix)
  oddsratios <- (x1 / (1 - x1)) / (x2 / (1 - x2))
  ormatrix[, i] <<- oddsratios
  contrast[i] <<- name
  or[i] <<- oddsratios[1]
  cilwr[i] <<- quantile(oddsratios, probs = 0.025, na.rm = T)
  ciupr[i] <<- quantile(oddsratios, probs = 0.975, na.rm = T)
  reps[i] <<- sum(!is.na(oddsratios))
  pvalue[i] <<- min(sum(oddsratios < 1), sum(oddsratios > 1)) * 2 / reps[i]
}

compareAnnualGenders <- function(i, index, ormatrix, name) {
  oddsratios <- ormatrix[, index + 6] / ormatrix[, index + 12]
  contrast[i] <<- name
  or[i] <<- oddsratios[1]
  cilwr[i] <<- quantile(oddsratios, probs = 0.025, na.rm = T)
  ciupr[i] <<- quantile(oddsratios, probs = 0.975, na.rm = T)
  reps[i] <<- sum(!is.na(oddsratios))
  pvalue[i] <<- min(sum(oddsratios < 1), sum(oddsratios > 1)) * 2 / reps[i]
```

```
}

compareAnnualT2 <- function(i, index, ormatrix, name) {
  oddsratios <- ormatrix[, index] / ormatrix[, index + 3]
  contrast[i] <<- name
  or[i] <<- oddsratios[1]
  cilwr[i] <<- quantile(oddsratios, probs = 0.025, na.rm = T)
  ciupr[i] <<- quantile(oddsratios, probs = 0.975, na.rm = T)
  reps[i] <<- sum(!is.na(oddsratios))
  pvalue[i] <<- min(sum(oddsratios < 1), sum(oddsratios > 1)) * 2 / reps[i]
}

compareAnnualAges <- function(i, responses, repetitions, name, c1, c2) {
  resp1 <- responses[, 9:16] # 1 year later
  resp2 <- responses[, 1:8]  # at start
  matrix <- matrix(rep(c1, repetitions), nrow = repetitions, byrow = T)
  x1 <- rowSums(resp1 * matrix)
  x2 <- rowSums(resp2 * matrix)
  matrix <- matrix(rep(c2, repetitions), nrow = repetitions, byrow = T)
  x3 <- rowSums(resp1 * matrix)
  x4 <- rowSums(resp2 * matrix)
  oddsratios <- ((x1 / (1 - x1)) / (x2 / (1 - x2))) / ((x3 / (1 - x3)) / (x4 / (1 - x4)))
  contrast[i] <<- name
  or[i] <<- oddsratios[1]
  cilwr[i] <<- quantile(oddsratios, probs = 0.025, na.rm = T)
  ciupr[i] <<- quantile(oddsratios, probs = 0.975, na.rm = T)
  reps[i] <<- sum(!is.na(oddsratios))
  pvalue[i] <<- min(sum(oddsratios < 1), sum(oddsratios > 1)) * 2 / reps[i]
}
```

```
  calculateAnnualOR(1, responses, repetitions, "Total T2DM"     , c(0,nf2,0,nf4,
0,nm2,0,nm4)/(nf2+nf4+nm2+nm4))
  calculateAnnualOR(2, responses, repetitions, "18-59 T2DM"     , c(0,nf2,0,0,
0,nm2,0,0)/(nf2+nm2))
  calculateAnnualOR(3, responses, repetitions, "60-xx T2DM"     , c(0,0,0,nf4,
0,0,0,nm4)/(nf4+nm4))
  calculateAnnualOR(4, responses, repetitions, "Total Nondiab"  , c(nf1,0,nf3,0,
nm1,0,nm3,0)/(nf1+nf3+nm1+nm3))
  calculateAnnualOR(5, responses, repetitions, "18-59 Nondiab"  , c(nf1,0,0,0,
nm1,0,0,0)/(nf1+nm1))
  calculateAnnualOR(6, responses, repetitions, "60-xx Nondiab"  , c(0,0,nf3,0,
0,0,nm3,0)/(nf3+nm3))

  calculateAnnualOR(7, responses, repetitions, "Total T2DM male"     , c(0,0,0,0,
0,nm2,0,nm4)/(nm2+nm4))
  calculateAnnualOR(8, responses, repetitions, "18-59 T2DM male"     , c(0,0,0,0, 0,1,0,0))
  calculateAnnualOR(9, responses, repetitions, "60-xx T2DM male"     , c(0,0,0,0, 0,0,0,1))
  calculateAnnualOR(10, responses, repetitions, "Total Nondiab male"  , c(0,0,0,0,
nm1,0,nm3,0)/(nm1+nm3))
  calculateAnnualOR(11, responses, repetitions, "18-59 Nondiab male"  , c(0,0,0,0, 1,0,0,0))
  calculateAnnualOR(12, responses, repetitions, "60-xx Nondiab male"  , c(0,0,0,0, 0,0,1,0))

  calculateAnnualOR(13, responses, repetitions, "Total T2DM female"     , c(0,nf2,0,nf4,
0,0,0,0)/(nf2+nf4))
  calculateAnnualOR(14, responses, repetitions, "18-59 T2DM female"     , c(0,1,0,0, 0,0,0,0))
  calculateAnnualOR(15, responses, repetitions, "60-xx T2DM female"     , c(0,0,0,1, 0,0,0,0))
  calculateAnnualOR(16, responses, repetitions, "Total Nondiab female"  , c(nf1,0,nf3,0,
0,0,0,0)/(nf1+nf3))
  calculateAnnualOR(17, responses, repetitions, "18-59 Nondiab female"  , c(1,0,0,0, 0,0,0,0))
  calculateAnnualOR(18, responses, repetitions, "60-xx Nondiab female"  , c(0,0,1,0, 0,0,0,0))
```

```
compareAnnualGenders(19, 1, ormatrix, "Total T2DM male-female")
compareAnnualGenders(20, 2, ormatrix, "18-59 T2DM male-female")
compareAnnualGenders(21, 3, ormatrix, "60-xx T2DM male-female")

compareAnnualGenders(22, 4, ormatrix, "Total Nondiab male-female")
compareAnnualGenders(23, 5, ormatrix, "18-59 Nondiab male-female")
compareAnnualGenders(24, 6, ormatrix, "60-xx Nondiab male-female")

compareAnnualT2(25, 1, ormatrix, "T2DM-Nondiab total")
compareAnnualT2(26, 2, ormatrix, "T2DM-Nondiab 18-59")
compareAnnualT2(27, 3, ormatrix, "T2DM-Nondiab 60-xx")

compareAnnualT2(28, 7, ormatrix, "T2DM-Nondiab total male")
compareAnnualT2(29, 8, ormatrix, "T2DM-Nondiab 18-59 male")
compareAnnualT2(30, 9, ormatrix, "T2DM-Nondiab 60-xx male")

compareAnnualT2(31, 13, ormatrix, "T2DM-Nondiab total female")
compareAnnualT2(32, 14, ormatrix, "T2DM-Nondiab 18-59 female")
compareAnnualT2(33, 15, ormatrix, "T2DM-Nondiab 60-xx female")

compareAnnualAges(34, responses, repetitions, "T2DM 18-59 - 60-xx"    , c(0,nf2,0,0, 0,nm2,0,0)/(nf2+nm2) , c(0,0,0,nf4,  0,0,0,nm4)/(nf4+nm4))
compareAnnualAges(35, responses, repetitions, "Nondiab 18-59 - 60-xx"    , c(nf1,0,0,0, nm1,0,0,0)/(nf1+nm1) , c(0,0,nf3,0,  0,0,nm3,0)/(nf3+nm3))
compareAnnualAges(36, responses, repetitions, "T2DM 18-59 - 60-xx male"    , c(0,0,0,0, 0,1,0,0) , c(0,0,0,0,  0,0,0,1))
compareAnnualAges(37, responses, repetitions, "Nondiab 18-59 - 60-xx male"    , c(0,0,0,0, 1,0,0,0) , c(0,0,0,0,  0,0,1,0))
compareAnnualAges(38, responses, repetitions, "T2DM 18-59 - 60-xx female"    , c(0,1,0,0, 0,0,0,0) , c(0,0,0,1,  0,0,0,0))
compareAnnualAges(39, responses, repetitions, "Nondiab 18-59 - 60-xx female"    , c(1,0,0,0, 0,0,0,0) , c(0,0,1,0,  0,0,0,0))
```

```
  response <- data.frame(cancer = cancers[cancerNumber], contrast = contrast, OR = or, 'CI lower' = cilwr, 'CI upper' = ciupr, 'p-value' = ifelse(round(pvalue, 4) < 0.0001, "<0.0001", round(pvalue, 4)), repetitions = reps)

  write.table(response, "years.csv", append = T, col.names = T, row.names = F, sep = ";", dec = ".")


})}


cancerNumber <- 0


# ----------------------------------------------------------------------------

(cancerNumber <- cancerNumber + 1)


# The number of new cancer cases by year, gender and age group from the SQL table for T2DM patients

cancerCasesDiab <- sqlQuery(channel, paste0("SELECT ev, nem, korcsop, betegszam FROM rxt_atzs_rd_diabinc1 where ALLAPOT = 'új diabéteszes' and DIABTIPUS = 'T2DM' and VISZONY = '1st year' and EV in ('2015', '2016', '2017', '2018') and NEM <> 'total' and KORCSOP in ('0-59', '60-xx') and CANCER_TYPE ='", cancers[cancerNumber], "'"), believeNRows=FALSE)

cancerCasesDiab$group <- 'T2DM'

cancerCasesDiab <- cancerCasesDiab %>% rename(year = EV, gender = NEM, age = KORCSOP, event = BETEGSZAM)


# The number of new cancer cases by year, gender and age group from the SQL table for non-diab patients

cancerCasesNonDiab <- sqlQuery(channel, paste0("SELECT ev, nem, korcsop, betegszam FROM rxt_atzs_rd_ccinc where DIABTIPUS = 'none' and VISZONY = 'free' and EV in ('2015', '2016', '2017', '2018', '2019') and NEM <> 'total' and KORCSOP in ('0-59', '60-xx') and CANCER_TYPE ='", cancers[cancerNumber], "'"), believeNRows=FALSE)

cancerCasesNonDiab$group <- 'Non-diab'

cancerCasesNonDiab <- cancerCasesNonDiab %>% rename(year = EV, gender = NEM, age = KORCSOP, event = BETEGSZAM)


cancerCases <- rbind(cancerCasesDiab, cancerCasesNonDiab)
```

```
cancerCases$gr <- paste(cancerCases$gender, cancerCases$age, cancerCases$group, sep='')
ds <- atRisk %>% left_join(cancerCases) %>% mutate(event = ifelse(is.na(event), 0, event), year2 = year - 2015)


model <- glm(cbind(event, nrisk) ~ gr + gr:year2 - 1, data = ds, family = "quasibinomial")
process_model(model)



# ---------------------------------------------------------------------------
(cancerNumber <- cancerNumber + 1)


# Similarly for the other cancers


# For breast, cervix, ovary and uterus we considered females only using a similar code
# For prostate and testis we considered males only using a similar code
```
